# Early rheumatoid arthritis, two distinctive structural damage patterns revealed by MRI: an 8-year longitudinal study

**DOI:** 10.1007/s00330-025-11493-5

**Published:** 2025-03-18

**Authors:** Su Wu, James Francis Griffith, Fan Xiao, Chungwun Yiu, Jason C. S. Leung, Lai-Shan Tam

**Affiliations:** 1 Department of Imaging and Interventional Radiology, The Chinese University of Hong Kong, Hong Kong, China; 2 Jockey Club Centre for Osteoporosis Care and Control, Faculty of Medicine, The Chinese University of Hong Kong, Hong Kong, China; 3 Department of Medicine & Therapeutics, Faculty of Medicine, The Chinese University of Hong Kong, Hong Kong, China

**Keywords:** Wrist, Rheumatoid arthritis, MRI, Follow-up study, Synovitis

## Abstract

**Objective:**

To determine how inflammatory and structural parameters change long-term on standard treatment in rheumatoid arthritis patients and which baseline parameter best predicts long-term structural damage.

**Material and methods:**

Prospective study of early rheumatoid arthritis (ERA) patients (symptom duration ≤ 24 months) who underwent identical clinical, serological, radiographic, and dynamic contrast-enhanced MRI of the wrist assessments at baseline, year-1, and year-8. MR images were analyzed semi-quantitatively (Rheumatoid Arthritis Magnetic Resonance Imaging Score [RAMRIS]) and quantitatively (synovial volume (cm^3^); synovial perfusion; bone marrow edema (BME) proportion [%]). Multivariate analyses and receiver operating curves were applied to find the best predictor of long-term structural damage.

**Results:**

81 patients (61 ± 12 years, F/M:67/14) were studied. MRI-detected inflammatory parameters markedly improved from baseline to year-1 and slightly deteriorated from year-1 to year-8 (synovial volume:6.7 ± 5.0→2.6 ± 2.9→3.6 ± 3.3 cm^3^ (*p* < 0.01); BME proportion:13.1 ± 9.3→7.4 ± 5.0→9.2 ± 9.7% [*p* < 0.01]). Structural damage progressively deteriorated from baseline to year-8. Two long-term structural damage pattern groups were apparent, namely a “non-progressive structural damage pattern” (62%, 50/81) and a “progressive structural damage pattern” (38%, 31/81). Functional impairment was more frequent and more severe at year-8 in patients with progressive structural damage. MRI-detected bone erosion score better predicted (AUC = 0.81, CI: 0.71–0.91) year-8 structural damage than clinical (SDAI AUC = 0.61, CI: 0.48–0.74), serological (CRP AUC = 0.60, CI: 0.47–0.73), or radiographic (AUC = 0.59, CI: 0.45–0.72) assessment.

**Conclusion:**

In ERA patients, two distinct structural damage patterns are evident. Baseline bone erosion score is better than clinical, serological, or radiographic assessment at predicting long-term structural damage.

**Key Points:**

***Questions*** *The value of MRI in predicting long-term structural damage in ERA patients is not clear.*

***Findings*** *This study identified two distinct long-term structural damage progression patterns of ERA patients. MRI can better differentiate between these two groups at baseline than clinical, serological, or radiographic assessment.*

***Clinical relevance*** *MRI examination should be performed in all ERA patients at baseline to determine their structural damage pattern. This will allow a better prediction of patient outcomes in the long-term.*

**Graphical Abstract:**

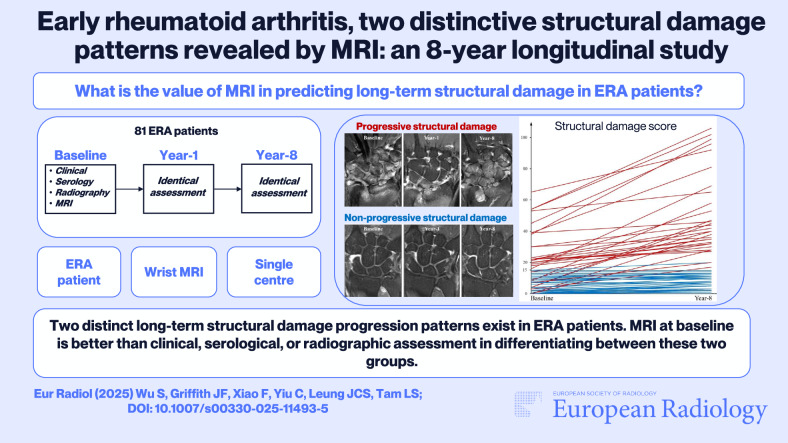

## Introduction

The goal of rheumatoid arthritis (RA) treatment is to minimize structural damage and preserve function. Despite considerable recent advances in treatment, one-third of early rheumatoid arthritis (ERA) patients (symptom duration ≤ 24 months) have a poor therapeutic response, which remains mechanistically unexplained [1–4]. MRI allows appreciation and measurement of both the inflammation (synovitis, tenosynovitis, osteitis) and structural damage (bone erosions, joint space narrowing) associated with RA much better than radiographs [5–8]. As no longitudinal MRI-based studies beyond two years have been performed, relatively little is known about long-term inflammatory and structural changes in ERA patients. To address this, we followed a cohort of ERA patients over an eight-year period.

The study aims were:

- To determine how inflammatory and structural parameters change over 8 years in ERA patients on standard treatment.
- To determine whether clinical, serological, radiographic, or MRI assessment (semi-quantitative or quantitative) at baseline ± year-1 can predict long-term structural damage and functional outcome.

## Methods

Following Institutional Ethics Committee approval, a cohort of treatment naïve ERA patients was prospectively studied, with signed consent obtained from all participants.

### Patients

**Fig. 1:**
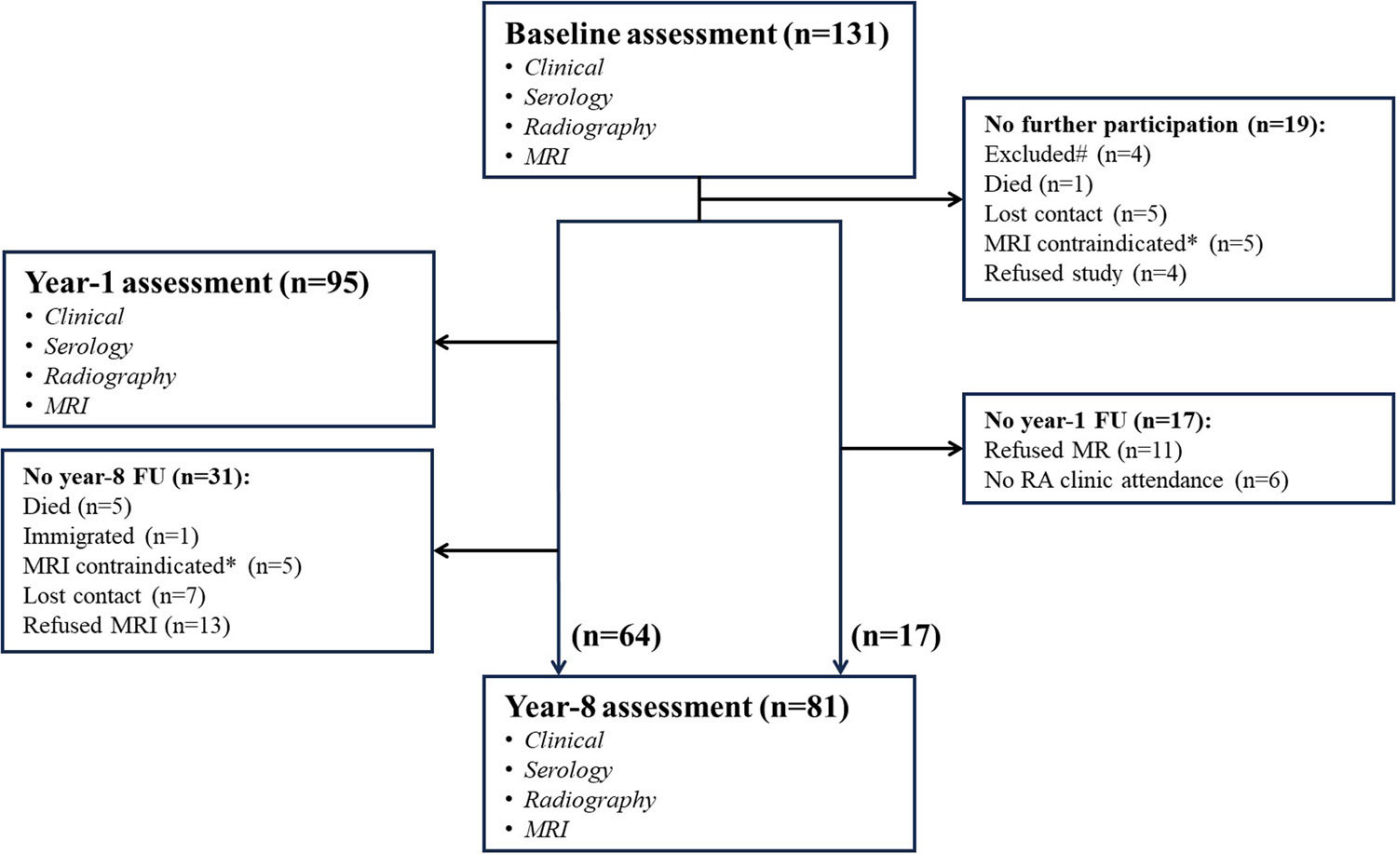
Flowchart of patient enrolment at baseline, year-1, and year-8. Sixty-four patients completed the baseline, year-1, and year-8 assessments. 17 patients did not undergo year-1 follow-up. #Excluded at year-1 due to diagnosis of systemic lupus erythematosus (*n* = 3) and peripheral spondylarthritis (*n* = 1). * Contrast enhanced MRI was contradicted because of implant (*n* = 3), claustrophobia (*n* = 1), malignant tumor (*n* = 4), kidney failure (*n* = 1), and age more than 90 years (*n* = 1). There was no significant difference in clinical, serological, radiographic, or MRI parameters at baseline between the 50 patients who did not complete and the 81 patients who did complete the year-8 follow-up (*p* > 0.05 for all)

ERA patients were recruited from the Rheumatology Clinic. The time interval between symptom onset and recruitment was 10.0 ± 6.7 months. Patients were treated-to-target according to a pre-specified treatment protocol aiming at remission. Treatment comprised methotrexate (7.5 mg/week) initially, which, depending on the disease activity, was increased to a maximum of 20 mg/week or the maximum tolerated dose. Prednisolone at a dose of ≤ 10 mg/day was allowed. Other conventional synthetic DMARDs (csDMARDs) were administered if patients failed to achieve the treatment target, occasionally followed by biologic DMARDs. Clinic visits were arranged every 3 months for the first 2 years and once a year thereafter. Patients underwent standard radiographs and DCE MRI at baseline and were re-called for radiographic and MRI assessments at year-1 and year-8 after initial presentation (Fig. 1). One-year follow-up in 77 of these patients has been published [9]. Patients who did not attend the year-8 follow-up were excluded from this study.

### Clinical, serological, and functional assessment

All clinical assessments were performed by the same rheumatology professor who has more than 20 years’ of diagnosing and treating RA. Disease activity was assessed using early morning stiffness duration, pain score, Simple Disease Activity Index (SDAI), Disease Activity Index 28 (DAS 28), physician global assessment (PGA), and patient global assessment (Pt GA). ESR and CRP were routinely measured. DAS remission is defined as DAS28 score < 2.6. SDAI remission was defined as SDAI score ≤ 3.3. Functional status was assessed using the Health Assessment Questionnaire—Disability Index (HAQ-DI), and functional impairment was defined as a single HAQ of > 0.5 or a HAQ increase of > 0.35 compared to year-1 [10].

### Radiographic assessment

Posteroanterior radiographs of both hands and wrists were scored chronologically using the Sharp-van der Heijde (SHS) method by one musculoskeletal radiologist with 3 years’ experience, blinded to clinical and MRI data [11]. This method semi-quantifies both joint space narrowing (JSN) (0–4 with 15 joints assessed) and bone erosion (0-5,16 bone areas assessed).

### MRI assessment

MRI of the most symptomatic wrist was scanned using an identical protocol. The scan protocol is outlined in Supplementary Material 1.

#### (A) MRI-based assessment of inflammatory activity

1. Semi-quantitative assessment. RAMRIS score of synovitis, tenosynovitis, and bone marrow edema (BME) was assessed chronologically by two musculoskeletal radiologists, one with 6 years, the other with 3 years of experience, both supervised by a musculoskeletal radiologist with 30 years of MR experience [12].
2. Quantitative assessment
  - Synovial volume. Joint synovitis and tenosynovitis volume (cm^3^) was measured on post-contrast T1-weighted fat-suppressed axial images. The area of synovitis and tenosynovitis was manually outlined by a single radiologist on serial consecutive images, and volume was automatically calculated by ITK-SNAP, a freely available segmentation tool, by summating all serial image areas [13]. It took 15 min to complete one wrist. (Supplementary Fig. 1a, b).
  - Synovial perfusion. Two perfusion parameters, maximum enhancement (Emax) and enhancement slope (Eslope) were measured [14]. A region of interest was manually placed on the largest area of synovial proliferation. Curve fitting was applied to the dynamic contrast-enhanced time-intensity plot using Prism (Version 9.0) software. (Supplementary Fig. 1c).
  - BME. A fully automated program was used to calculate the overall proportion (%) of the wrist bone areas affected by BME as well as the relative intensity on T2-weighted fat-suppressed coronal images (Supplementary Figure 1d) [15]. It took 3 min to complete one wrist.

#### (B) MRI-based assessment of structural damage

Structural damage was semi-quantitatively assessed utilizing RAMRIS scoring of bone erosion and JSN to yield an overall “structural damage score”, which was the summation of bone erosion and JSN scores.

**Fig. 2:**
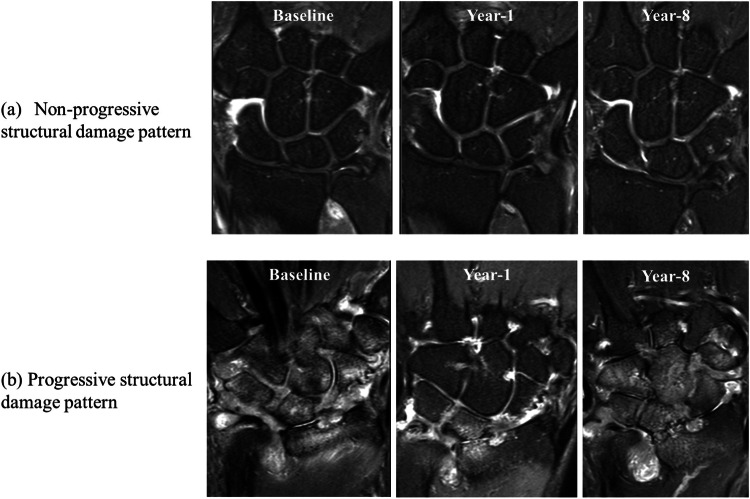
Two structural damage progression patterns at baseline, year-1, and year-8. **a** Non-progressive structural damage group: 50-year-old female T2FS coronal MR images of wrist. Structural damage score (bone erosion+ JSN) was 1 at baseline, 1 at year-1, and 2 at year-8. **b** Progressive structural damage group; 51-year-old female T2FS coronal MR images of wrist. Structural damage score was 22 at baseline, 25 at year-1, and 69 at year-8

1. Bone erosion. Bone erosions were scored on T1- and T2-weighted fat-suppressed coronal and axial images with erosion site and size drawn on a schematic wrist bone template (Supplementary Fig. 2). Bone erosion score was based on the proportion of bone area eroded: 0: no erosion; 1: < 10% bone area eroded, 2: < 20% bone area eroded, etc. If adjacent bones are fused, both bones were scored as 10. Ten wrist bones and the base of five metacarpal bones were scored.
2. JSN. JSN was graded at 17 joints spaces from 0–4 as follows: no JSN: 0, mild JSN:1, moderate JSN:2, severe JSN: 3, and ankylosis:4.
3. Structural damage score and smallest detectable change (SDC). Utilizing a recognized analytic method [16], the SDC of structural damage score in this study is 6.61. As such, a structural damage score (bone erosion and JSN) of > 6.61 between baseline and year-8 was categorized as “progressive structural damage” while < 6.61 was categorized as “non-progressive structural damage” (Fig. 2).

### Reliability of MR RAMRIS analysis

Both readers, blinded to clinical assessment, independently scored all baseline and year-1 cases (*n* = 145) for all measured parameters, year-8 was scored by one reader (*n* = 81). 30 randomly selected image databases were re-scored by both readers four weeks after the initial scoring.

### Statistical analysis

SPSS software (version 20.0, IBM) was used for data analysis. Repeated measurements of ANOVA and Friedman’s test were used to test differences between clinical and MRI measurements at different time points. Post-hoc analysis with Bonferroni correction was performed to determine group differences. Intergroup differences were assessed using independent Student *t*-test or Mann-*U*-test for quantitative variables, and chi-squared test for categorical variables. *p*-value < 0.05 was considered statistically significant.

For semi-quantitative assessment, inter- and intra-class correlation test (ICC) was used to test reliability. ICC ≤ 0.2 indicated poor agreement; 0.21–0.40, fair agreement; 0.41–0.60, moderate agreement; 0.61–0.80, substantial agreement; and 0.81–1.00 excellent agreement. Scores of both researchers were averaged before analysis.

Univariable and multivariable logistic regression analysis was performed to assess the relationship between baseline and year-1 radiographic and MRI parameters and year-8 structural damage. Receiver operating characteristic (ROC) analysis was used to determine the optimal cut-off value and assess the predictive value of clinical, serological, radiological, and MRI features at baseline for determining structural damage severity at year-8. The area under the curve (AUC) of different factors was also compared, AUC curve values of < 0.7 were considered poor, 0.7–0.8 as moderate, and > 0.8 as good. The Youden Index was used to find the optimal cut-off value for structural damage score on baseline MRI to differentiate between progressive and non-progressive structural damage groups.

## Results

### Patient characteristics

Time interval between baseline and last MRI examination was 8.2 ± 1.9 years. The interval between clinical and MRI assessments was 15.6 ± 14.4 days. Patient characteristics, as well as clinical and MRI assessment data, are shown in Table 1. At year-8, most patients were on conventional DMARDs. More patients were on biologic DMARDs at year-8 than year-1 with a small percentage of patients still on prednisolone. More patients achieved clinical remission at year-8 than year-1 though this did not reach statistical significance (Table 1).

**Table 1:** Patient characteristics, serological, radiographic, and MRI assessment results at baseline, year-1, and year-8

	Baseline	Year-1	Year-8	*p-*value
	(*n* = 81)	(*n* = 64)	(*n* = 81)	
Age (years)	53.4 ± 12.6	56.2 ± 12.6	61.3 ± 12.3	**<** **0.001**
Sex (F/M)	67/14	52/12	67/14	0.819
RF positive (%)	69 (85.2)	—	—	—
Anti-CCP IgM positive (%)	72 (88.9)	—	—	—
Conventional DMARDs (%)	—	58 (93.5)	69 (85.2)	0.324
Biologic DMARDs (%)	—	4 (6.3)	18 (22.2)	**0.008**
Oral prednisolone (%)	—	14 (21.9)	6 (7.4)	**0.012**
Clinical assessments				
Early morning stiffness (min)	60 (0–180)	0 (0–30)	0 (0–10)	**<** **0.001**
Pain score	5.2 ± 2.2	2.5 ± 2.1	2.4 ± 2.4	**<** **0.001**
Patient global assessment	6.0 ± 2.0	2.4 ± 2.5	2.7 ± 2.2	**<** **0.001**
Physician global assessment	6.0 ± 2.5	2.3 ± 2.5	1.9 ± 2.1	**<** **0.001**
Tender joint count	7 (4–11)	3 (0–4)	1 (0–3)	**<** **0.001**
Swollen joint count	4 (2–7)	0 (0–2)	0 (0–1)	**<0.001**
DAS28	4.8 ± 1.1	2.6 ± 1.1	2.5 ± 1.1	**<** **0.001**
DAS28 remission^a^ (%)	0	34 (53.1)	63 (77.8)	0.727
SDAI	25.6 ± 10.6	8.6 ± 9.7	7.9 ± 7.6	**<** **0.001**
SDAI remission^b^ (%)	0	24 (37.5)	31 (38.3)	0.924
HAQ	0.9 ± 0.7	0.3 ± 0.5	0.4 ± 0.4	**<** **0.001**
Acute phase reactants				
ESR	49 (30–81)	34 (18–47)	26 (18–44)	**<** **0.001**
CRP	8.9 (2.4–26.0)	2.4 (0.7–6.1)	1.7 (0.7–6.5)	**<** **0.001**
Radiographic assessment				
SHS score	2.2 ± 4.4	3.9 ± 6.5	6.3 ± 8.9	**<** **0.001**
MRI semi-quantitative assessment				
Synovitis score	5.9 ± 3.1	3.5 ± 2.5	4.1 ± 2.7	**<** **0.001**
Tenosynovitis score	3.9 ± 3.6	1.4 ± 2.0	1.7 ± 2.3	**<** **0.001**
Bone marrow edema score	7.2 ± 8.4	2.2 ± 3.6	3.1 ± 6.2	**<** **0.001**
Bone erosion score	9.4 ± 9.6	10.1 ± 12.3	14.3 ± 17.4	**<** **0.001**
JSN score	3.3 ± 7.0	3.3 ± 7.2	7.4 ± 10.8	**<** **0.001**
Total score	29.4 ± 23.8	20.8 ± 23.7	30.7 ± 33.8	**<** **0.001**
MRI quantitative assessment				
Synovitis volume (cm^3^)	5.5 ± 4.4	2.4 ± 2.7	3.2 ± 3.0	**<** **0.001**
Tenosynovitis volume (cm^3^)	1.2 ± 1.5	0.2 ± 0.5	0.4 ± 0.7	**<** **0.001**
Total volume (cm^3^)	6.7 ± 5.0	2.6 ± 2.9	3.6 ± 3.3	**<** **0.001**
Emax (%)	70.1 ± 38.9	42.0 ± 45.7	47.7 ± 41.1	**<** **0.001**
Eslope (%/s)	6.3 ± 6.9	2.9 ± 4.8	2.7 ± 5.2	**<** **0.001**
Bone marrow edema (%)	13.1 ± 9.3	7.4 ± 5.0	9.2 ± 9.7	**<** **0.001**

Dichotomous variables are presented as *n* (%), normally distributed data are mean ± standard deviation, and skewed data as median (interquartile range). — not available, *RF* rheumatoid factor, *anti-CCP* anti-cyclic citrullinated peptide, *DAS* disease activity score

Statistically significant values (*p* < 0.05) are bolded

^a^ Defined as DAS28 < 2.6. *SDAI* Simple Disease Activity Index

^b^ Defined as SDAI ≤ 3.3. *DMARDs* disease-modifying antirheumatic drugs, *HAQ* Health Assessment Questionnaire, *ESR* erythrocyte sedimentation rate, *CRP* C-reactive protein, *SHS* score Sharp-van der Heijde score, *JSN* joint space narrowing, *Emax* maximum enhancement, *Eslope* enhancement slope

#### Reliability of MR analysis

RAMRIS reliability was excellent for synovitis, BME, and bone erosion scores, with an inter-reader ICC of 0.883-0.941 and intra-reader ICC of 0.900-0.961. (Supplementary Table 1).

### Clinical and serological parameters at baseline, year-1, and year-8

All clinical parameters improved markedly by year-1 (Table 1). Between year-1 and year-8, clinical parameters remained unchanged or slightly deteriorated while ESR and CRP continued to decrease from baseline to year-8 (Table 1). Post-hoc analysis showed clinical and serological parameters significantly improved between baseline and year-1 with no significant difference between year-1 and year-8 (Table 1).

### MRI inflammatory parameters at baseline, year-1, and year-8

(i) Semi-quantitative assessment. All semi-quantitative parameters (synovitis, tenosynovitis, BME) decreased markedly by year-1 (Table 1). Between year-1 and year-8, MRI inflammatory parameters increased slightly, though remained below baseline values at year-8 (Table 1).
(ii) Quantitative assessment
  - Synovial volume. Synovial volume markedly decreased from 5.5 ± 4.4 cm^3^ at baseline to 2.4 ± 2.7 cm^3^ at year-1 (Table 1). Between year-1 and year-8, synovial volume slightly increased (to 3.2 ± 3.0 cm^3^) (Table 1). Similarly, compared to baseline, teno-synovial volume decreased markedly by year-1 and then increased slightly by year-8. (Table 1)
  - Synovial perfusion. Emax decreased markedly from 70.1 ± 38.9% at baseline to 42.0 ± 45.7% at year-1, and then slightly increased to 47.7 ± 41.1% at year-8. Eslope continued to decrease from baseline to year-8 (Table 1).
  - BME. BME proportion decreased from 13.1 ± 9.3% at baseline to 7.4 ± 5.0% at year-1 and then increased slightly to 9.2 ± 9.7% at year-8 (Table 1).

### Radiographic and MRI structural damage at baseline, year-1, and year-8

**Fig. 3:**
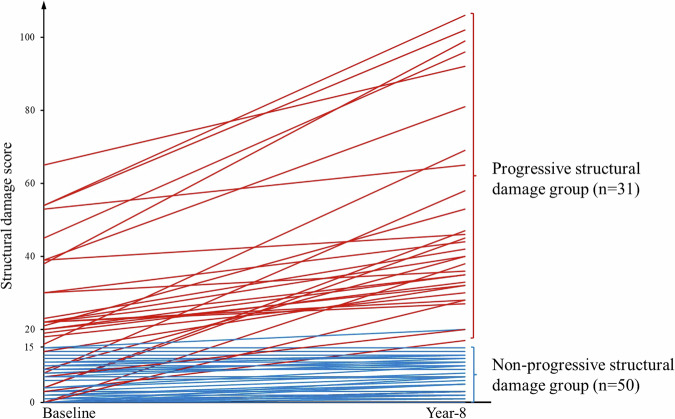
Case-by-case representation of structural damage score from baseline to year-8 in 81 ERA patients. Red lines represent patients (*n* = 31, 38%) patients with a “progressive structural damage pattern”. Blue lines represent patients (*n* = 50, 62%) with a “non-progressive structural damage pattern”. A structural damage score of 15 (actual value 14.5) was the optimal Youden Index value for differentiating between non-progressive and progressive structural damage groups at baseline
  In the non-progressive structural damage group (*n* = 50), all patients had none-mild structural damage at baseline with a structural damage score of 6.8 ± 7.8. This subgroup of patients showed little or no progression in structural damage, with a structural damage score at year-8 of 7.0 ± 7.5.
  In the progressive structural damage group (*n* = 31), 24 (77%) of 31 patients had moderate-severe structural damage at baseline with a structural damage score of 23.0 ± 18.4. Seven (23%) of the 31 patients had none-mild structural damage (structural score < 15) at baseline but significantly deteriorated thereafter (Fig. 3) (Table 2). MRI-based inflammatory and structural parameters, whether assessed semi-quantitatively or quantitatively, were higher in this progressive structural damage group than the non-progressive group at baseline, year-1, and year-8 (Table 2) (Fig. 4a–d).

- Radiographic structural damage. SHS score at baseline (2.2 ± 4.4) increased to 3.9 ± 6.5 at year-1 and to 6.3 ± 8.9 at year-8 (*p* < 0.001) (Table 1). At baseline, 75 (93%) of 81 patients had none or mild radiographic structural damage while at year-8, 61 (75%) of these 81 patients still had none or mild radiographic structural damage. Conversely, 6 (7%) and 20 (25%) of 81 patients had moderate or severe radiographic structural damage at baseline and year-8, respectively.
- MRI structural damage. Bone erosion and JSN deteriorated slightly between baseline and year-1 and continued to deteriorate between year-1 and year-8 (Table 1). Post-hoc analysis showed no significant difference in structural damage parameters between baseline and year-1, while at year-8, more severe structural damage was present than both baseline and year-1 (Table 1). Bone erosion increased from 9.4 ± 9.6 at baseline to 14.3 ± 17.4 at year-8 while JSN increased from 3.3 ± 7.0 at baseline to 7.4 ± 10.8 at year-8. Four (5%) of 81 patients had developed ankylosis by year-8.
- Structural damage progression groups. Two distinctive structural damage progressive pattern groups were apparent on MRI, namely a “non-progressive structural damage group” and a “progressive structural damage group” (Figs. 2 and 3). At year-8, of 81 patients, 50 (62%) patients belonged to the non-progressive structural damage group, while 31 (38%) belonged to the progressive structural damage group. The average time interval between symptom-onset and baseline MRI examination was similar for both groups (9.8 vs. 10.4 months, *p* = 0.71). The optimal cut-off value for structural damage score at baseline was 14.5 (for practical purposes, 14.5 was taken as 15) with a sensitivity of 58% and specificity of 88%.

**Table 2:** MRI-detected parameters (semi-quantitative and quantitative) in non-progressive and progressive structural damage groups at baseline, year-1, and year-8

	Baseline			Year-1			Year-8		
	Non-progressive group	Progressive group	*p-*value	Non-progressive group	Progressive group	*p-*value	Non-progressive group	Progressive group	*p-*value
	(*n* = 50)	(*n* = 31)		(*n* = 41)	(*n* = 23)		(*n* = 50)	(*n* = 31)	
MRI semi-quantitative assessment									
Synovitis (RAMRIS)	5.1 ± 3.1	7.3 ± 2.6	**0.001**	2.7 ± 1.8	4.9 ± 3.1	**0.004**	2.8 ± 1.9	6.0 ± 2.7	**<** **0.001**
Tenosynovitis (RAMRIS)	3.7 ± 3.6	4.2 ± 3.7	0.517	1.3 ± 2.0	1.6 ± 2.2	0.804	1.3 ± 2.2	2.3 ± 2.4	**0.012**
BME (RAMRIS)	5.0 ± 6.3	10.7 ± 10.1	**0.008**	1.0 ± 1.9	4.3 ± 4.9	**0.001**	1.1 ± 4.1	6.5 ± 7.4	**<** **0.001**
Bone erosion (RAMRIS)	5.6 ± 6.0	15.6 ± 11.2	**<** **0.001**	5.3 ± 6.4	18.4 ± 15.4	**<** **0.001**	5.8 ± 5.5	28.6 ± 20.5	**<** **0.001**
Δ bone erosion,	—	—	—	1.1 ± 2.7	5.2 ± 7.9	**0.026**	1.1 ± 1.8	15.5 ± 13.2	**<** **0.001**
JSN (RAMRIS)	1.1 ± 2.5	6.9 ± 9.9	**0.003**	1.0 ± 2.4	7.7 ± 10.4	**0.002**	1.2 ± 3.0	17.4 ± 11.3	**<** **0.001**
Δ JSN	—	—	—	0.4 ± 2.0	1.4 ± 3.1	0.124	0.5 ± 1.0	11.5 ± 8.6	**<** **0.001**
Total score (RAMRIS)	20.6 ± 16.4	43.9 ± 27.0	**<** **0.001**	11.3 ± 11.2	38.8 ± 30.3	**<** **0.001**	12.0 ± 12.4	60.8 ± 35.8	**<** **0.001**
MRI quantitative assessment									
Synovitis volume (cm^3^)	4.5 ± 4.4	7.1 ± 4.0	**0.011**	1.7 ± 1.7	3.4 ± 3.6	**0.039**	1.9 ± 1.6	4.9 ± 3.7	**<** **0.001**
Tenosynovitis volume (cm^3^)	1.2 ± 1.5	1.2 ± 1.5	0.902	0.2 ± 0.7	0.2 ± 0.3	0.586	0.3 ± 0.6	0.5 ± 0.7	0.217
Total volume (cm^3^)	5.7 ± 5.3	8.3 ± 4.5	**0.027**	2.0 ± 2.3	3.6 ± 3.6	**0.035**	2.2 ± 2.0	5.4 ± 3.8	**<** **0.001**
BME proportion (%)	10.1 ± 6.7	18.0 ± 10.8	**0.001**	5.6 ± 2.8	10.4 ± 6.3	**0.001**	5.7 ± 2.6	16.7 ± 13.4	**<** **0.001**
Emax (%)	69.8 ± 32.9	76.3 ± 25.8	0.441	39.7 ± 50.7	45.9 ± 32.6	0.679	35.8 ± 39.0	74.1 ± 33.0	**0.001**
Eslope (%/s)	10.1 ± 6.6	18.8 ± 18.4	**0.049**	2.7 ± 5.2	3.3 ± 3.8	0.723	1.5 ± 3.0	5.3 ± 7.7	**0.009**

Dichotomous variables are presented as *n* (%), normally distributed data is presented as mean ± SD, and skewed data as median (interquartile range). —: not available, Δ: compared with baseline. *BME* bone marrow edema, *JSN* joint space narrowing, *Emax* maximum enhancement, *Eslope* enhancement slope

Statistically significant values (*p* < 0.05) are bolded

**Fig. 4:**
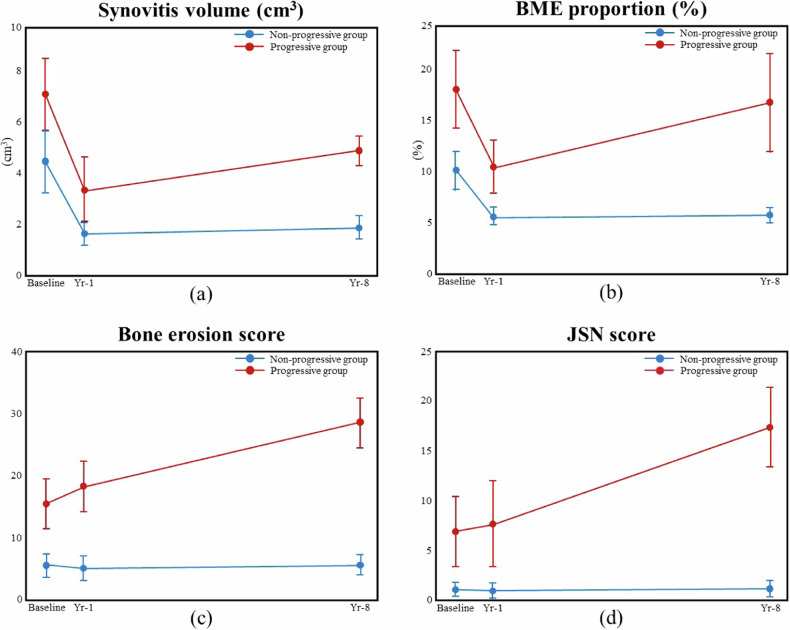
Inflammatory and structural damage parameters in the non-progressive structural damage group (blue line) and progressive structural damage group (red line) at baseline, year-1 and year-8. Mean and 95% confidence interval are depicted. **a** Synovial volume (cm^3^). **b** Bone marrow edema proportion (%). **c** Bone erosion score, and **d** Joint space narrowing score

### Baseline and year 1 predictors of structural damage progression at year-8

Patient age, sex, RF factor, anti-CCP IgM, ESR, and CRP were included in the multivariate prediction model as covariates. In the multivariable logistic regression analysis, the standard deviation of each factor was considered as one unit (Table 3). Bone erosion, JSN, and RAMRIS synovitis score at baseline and year-1 were related to structural damage progression at year-8 in univariate and multivariate analysis, with a greater OR for bone erosion (5.61) than JSN (4.84) or synovitis RAMRIS score (2.71).

**Table 3:** Relative risk (odds ratio) of baseline radiographic and MRI parameters for predicting structural damage severity at year-8

	Univariate	*p-*value	Multivariate^a^	*p*-value
	OR (95% CI)^b^		OR (95% CI)^b^	
Radiographic parameter				
SHS score	2.79 (1.08 to 7.20)	**0.035**	1.74 (0.99 to 3.06)	0.054
MRI semi-quantitative parameters				
Synovitis score	2.24 (1.32 to 3.79)	**0.003**	2.17 (1.15 to 4.08)	**0.008**
Tenosynovitis score	1.16 (0.74 to 1.83)	0.512	1.02 (0.58 to 1.78)	0.948
BME	2.09 (1.24 to 3.53)	**0.007**	1.89 (1.05 to 3.41)	**0.034**
Bone erosion	3.67 (1.90 to 7.12)	**<** **0.001**	4.05 (1.90 to 8.62)	**<** **0.001**
JSN	3.47 (1.45 to 8.30)	**0.006**	3.24 (1.32 to 7.95)	**0.011**
MRI quantitative parameters				
Synovitis volume (cm^3^)	1.65 (1.00 to 2.71)	**0.049**	1.78 (1.01 to 3.13)	**0.044**
BME proportion (%)	2.16 (1.21 to 3.88)	**0.001**	1.85 (0.96 to 3.50)	0.050
Emax (%)	1.26 (0.59 to 2.71)	0.563	1.48 (0.55 to 3.98)	0.449
Eslope (%/s)	1.01 (0.99 to 1.04)	0.481	1.02 (0.98 to 1.06)	0.331

*CI* confidence interval, *SHS score* Sharp-van der Heijde score, *BME* bone marrow edema, *JSN* joint space narrowing, *Emax* maximum enhancement, *Eslope* enhancement slope. Patients were not on treatment at baseline. Bone erosion, followed by JSN, and synovitis score were the best baseline predictors of structural damage severity at year-8

Statistically significant values (*p* < 0.05) are bolded

^a^ For multivariate regression analysis, patient age, sex, RF factor, anti-CCP IgM, ESR, and CRP were considered as covariates

^b^ Standard deviation of each factor was considered as one unit in logistic regression analysis

In univariable and multivariable analysis, patient treatment in year-1 (Table 4) (conventional and biological DMARDs) was not related to long-term structural damage progression.

**Table 4:** Relative risk (odds ratio) of year-1 treatment, radiographic and MRI parameters for predicting structural damage severity at year-8

	Univariate	*p*-value	Multivariate^a^	*p*-value
	OR (95% CI)^b^		OR (95% CI)^b^	
Year-1 treatment				
Conventional DMARDs	0.49 (0.06 to 3.73)	0.489	0.32 (0.03 to 2.94)	0.314
Biologic DMARDs	2.74 (0.37 to 20.48)	0.327	3.06 (0.19 to 9.65)	0.432
Oral prednisolone	3.11 (0.92 to 10.53)	0.068	3.39 (0.73 to 5.74)	0.120
Radiographic parameter				
SHS score	2.58 (1.42 to 4.70)	**0.002**	3.14 (1.36 to 7.27)	**0.008**
MRI semiquantitative parameter				
Synovitis	2.69 (1.42 to 5.09)	**0.002**	2.71 (1.32 to 5.59)	**0.007**
Tenosynovitis	1.11 (0.76 to 1.63)	0.591	1.58 (0.84 to 2.98)	0.159
BME	3.32 (1.45 to 7.57)	**0.005**	4.67 (1.49 to 14.63)	**0.008**
Bone erosion	3.76 (1.77 to 7.98)	**0.001**	5.61 (1.28 to 24.65)	**0.022**
JSN	3.68 (1.43 to 9.48)	**0.007**	4.84 (1.19 to 19.62)	**0.027**
MRI quantitative parameter				
Synovitis volume (cm^3^)	1.98 (1.08 to 3.62)	**0.026**	2.00 (0.89 to 4.46)	0.092
BME proportion (%)	3.11 (1.52 to 6.36)	**0.002**	3.39 (1.39 to 8.26)	**0.008**
Emax (%)	3.01 (1.14 to 7.93)	**0.026**	1.62 (0.80 to 3.27)	0.176
Eslope (%/s)	1.09 (0.96 to 1.23)	0.178	1.08 (0.97 to 1.20)	0.139

*DMARDs* disease-modifying antirheumatic drugs, *SHS score* Sharp-van der Heijde score, *BME* bone marrow edema, *JSN* joint space narrowing, *Emax* maximum enhancement, *Eslope* enhancement slope. Bone erosion, followed by JSN and BME were the best year-1 predictors of structural damage severity at year-8

Statistically significant values (*p* < 0.05) are bolded

^a^ In multivariate regressive analysis, patient age, sex, RF factor, anti-CCP IgM, ESR, and CRP were considered as covariates

^b^ Standard deviation of each factor was considered as one unit in logistic regression analysis

**Fig. 5:**
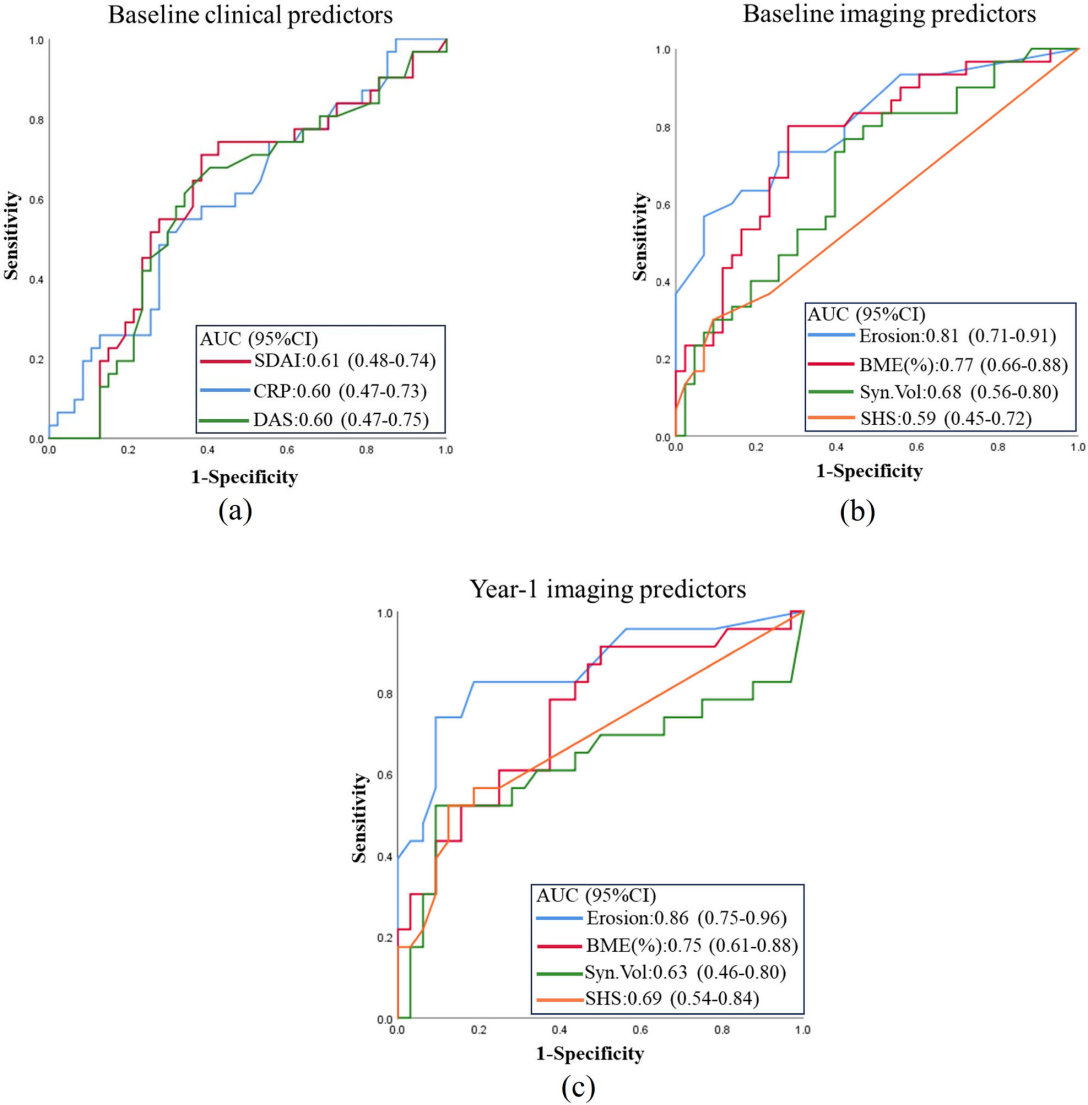
Receiver operating characteristics (ROC) curve in predicting structural damage progression at year-8. **a** Predictive capacity of baseline clinical parameters. **b** Predictive capacity of baseline MRI parameters and SHS radiographic score. **c** Predictive capacity of year-1 MRI parameters and SHS radiographic score. AUC, area under the curve; SDAI, Simple Disease Activity Index; DAS, disease activity score; BME, bone marrow edema; CRP, C-reactive protein; Syn. Vol, synovitis volume; SHS, Sharp-van der Heijde score

All baseline clinical, serological, and radiographic parameters were poor predictors of structural damage at year-8 (Fig. 5a). All baseline MR parameters were better predictors of structural damage at year-8 (Fig. 5b), with baseline MR bone erosion score being the best predictor (AUC = 0.81) followed by BME (AUC = 0.77) (Fig. 5b). The predictive capacity for both MRI bone erosion score and SHS score was slightly better at year-1 than baseline (Fig. 5c).

**Table 5:** Clinical parameters of non-progressive and progressive structural damage groups at baseline, year-1, and year-8

	Baseline			Year-1			Year-8		
	Non-progressive group	Progressive group	*p-*value	Non-progressive group	Progressive group	*p-*value	Non-progressive group	Progressive group	*p-*value
	(*n* = 50)	(*n* = 31)		(*n* = 41)	(*n* = 23)		(*n* = 50)	(*n* = 31)	
Age (years):	53.9 ± 11.4	50.4 ± 13.3	0.210	56.2 ± 11.6	53.4 ± 12.7	0.433	62.4 ± 11.3	59.0 ± 13.7	0.291
Sex (F/M):	42/8	25/6	0.698	35/6	17/6	0.260	42/8	25/6	0.698
RF positive *n* (%)	43 (86.0)	26 (83.9)	0.516	—	—	—	—	—	—
Anti-CCP IgM positive (%)	42 (84.0)	30 (96.8)	0.073	—	—	—	—	—	—
Conventional DMARDs (%)	—	—	—	39 (95.1)	19 (90.5)	0.481	41 (82.0)	28 (90.3)	0.305
Biologic DMARDs (%)	—	—	—	2 (4.9)	2 (8.7)	0.292	13 (26.0)	5 (16.1)	0.299
Oral prednisolone (%)	—	—	—	6 (14.6)	8 (34.8)	0.061	2 (4.0)	4 (12.9)	0.137
Clinical assessment									
Pain score	5.1 ± 2.4	5.4 ± 1.8	0.562	2.6 ± 2.3	2.2 ± 1.9	0.511	2.4 ± 2.6	2.5 ± 2.2	0.822
Patient global score	6.1 ± 2.1	6.0 ± 1.9	0.830	2.6 ± 2.4	2.2 ± 1.7	0.577	2.7 ± 2.3	2.7 ± 2.1	0.997
Physician global score	5.7 ± 2.4	6.5 ± 2.7	0.156	2.0 ± 2.2	2.7 ± 2.7	0.267	1.7 ± 2.1	1.9 ± 1.9	0.593
Tender joint count	7 (5–11)	8 (6–11)	0.332	3 (0–6)	2 (0–8)	0.954	1 (0–3)	1 (0–2)	0.771
Swollen joint count	4 (2–6)	5 (3–7)	0.107	0 (0–2)	1 (0–2)	0.186	0 (0–0)	0 (0–2)	**0.016**
DAS28	4.7 ± 1.1	4.9 ± 1.0	0.387	2.5 ± 1.1	2.8 ± 1.3	0.427	2.5 ± 1.1	2.5 ± 1.0	0.935
DAS28 remission^a^ (%)	0	0	—	24 (58.5)	10 (43.5)	0.247	35 (70.0)	17 (54.8)	0.167
SDAI	26.3 ± 12.3	30.0 ± 11.7	0.204	7.8 ± 8.0	10.1 ± 12.3	0.346	7.7 ± 8.1	8.4 ± 7.2	0.713
SDAI remission^b^ (%)	0	0	—	15 (36.6)	9 (39.1)	0.840	25 (50.0)	9 (29.0)	0.063
HAQ	1.0 ± 0.7	0.9 ± 0.6	0.760	0.3 ± 0.4	0.4 ± 0.6	0.927	0.3 ± 0.4	0.6 ± 0.5	**0.033**
Function impairment (%)	—	—	—	—	—	—	11 (22.0)	15 (48.4)	**0.026**
Acute phase reactants									
ESR	44 (28–70)	43 (33–80)	0.701	29 (16–46)	28 (14–48)	0.817	26 (18–44)	26 (21–49)	0.477
CRP	7.2 (1.7–21.1)	14.0 (6–36)	0.175	1.0 (0.6–3)	1.9 (0.7–9.4)	0.143	1.1 (0.6–4.6)	2.7 (0.9–7.5)	0.076

Dichotomous variables are presented as *n* (%), normally distributed data is presented as mean ± standard deviation, and skewed data is shown as median (interquartile range). — not available. *RF* rheumatoid factor, *anti-CCP* anti-cyclic citrullinated peptide, *DMARDs* disease-modifying antirheumatic drugs, *DAS* disease activity score

Statistically significant values (*p* < 0.05) are bolded

^a^ Defined as DAS28 <  2.6. *SDAI* Simple Disease Activity Index

^b^ Defined as SDAI ≤ 3.3. *HAQ* Health Assessment Questionnaire, *ESR* erythrocyte sedimentation rate, *CRP* C-reactive protein

### Relationship between disease remission and structural damage progression

Achieving DAS remission (*p* = 0.25) or SDAI remission (*p* = 0.84) at year-1 did not affect the likelihood of structural damage progression at year-8 (Table 5).

### Function loss in “non-progressive structural damage” and “progressive structural damage” groups

Functional impairment at year-8 was twice as common in the progressive structural damage group affecting 15 of 31 (48%) patients compared to 11 (22%) of 50 patients in non-progressive structural damage group. Functional impairment score was also twice as severe in the progressive structural damage group than the non-progressive structural damage group (Table 5).

## Discussion

The wrist is the most affected joint in ERA and a good marker of systemic inflammatory activity [17–19]. No prior long-term MRI-based ERA study has been performed, with the longest reported duration of MRI follow-up being two years [20]. The current study shows that over an eight-year period, clinical and serological inflammatory parameters continued to improve while MRI-based inflammatory parameters improved between baseline and year-1 and slightly deteriorated between year-1 and year-8. Radiographic and MRI-based structural damage scores continued to deteriorate from baseline to year-8.

MRI is much more sensitive and specific than radiographs at detecting structural damage [5–8]. The most notable finding of this study was the recognition on MRI of two distinct structural damage subgroups. The first group, comprising 62% of the cohort, had none-mild structural damage at baseline and continued to have none-mild structural damage by 8 years. The second group, comprising 38% of the cohort, had moderate-severe structural damage. Three-quarters of this second group had moderate to severe structural damage evident even at baseline and continued to deteriorate over 8 years. The remaining one-quarter had little or no structural damage at baseline though significantly deteriorated up to year-8. On average, patients with progressive structural damage had more severe synovitis, BME, bone erosion, and JSN than patients with non-progressive structural damage throughout the study period.

At year-8, functional outcome was twice as impaired and twice as common in the progressive structural damage group than in the non-progressive structural damage group [21, 22]. All other clinical assessments were similar between both groups. This is as expected since structural damage, particularly bone erosion, tends to induce disability rather than pain [23].

The results of this MR-based study help explain previous radiographic-based studies showing how some ERA patients had more rapid and severe progression than other patients [24–26] and how some ERA patients remain erosive-free even after 11 years [27]. It also helps explain why some patients continue to have structural damage progression even after they achieve clinical remission [28–30].

The recognition of two structural damage patterns in ERA concurs well with the recent finding of two distinctive ERA phenotypes based on synovial composition [31, 32]. Synovial biopsy revealed that 35-40% of ERA patients have a “B-cell rich synovitis” while 49-65% have “B-cell poor synovitis” [31, 32]. ERA patients with B-cell-rich synovitis have more inflammation and higher disease activity than patients with B-cell poor synovitis [31, 32]. The relationship between these two synovial phenotypes and the two structural MRI phenotypes described in this study is unknown. Different structural damage phenotypes may help explain the poor therapeutic response seen in one-third of ERA patients and provide a target for patient-centered treatment [32].

Only baseline MRI parameters, and not clinical, serological, or radiographic parameters, were helpful in predicting structural damage progression at year-8. Baseline MRI erosion is known to best predict radiographic progression [17, 28, 33–36]. Most patients with progressive structural damage at year-8 had clear structural damage already evident at baseline, suggesting the existence of one ERA subgroup prone to structural damage from the onset and another ERA subgroup never prone to develop structural damage despite ongoing inflammation. Although patients with progressive structural damage had more severe synovitis, the severity of baseline erosions was a much better long-term predictor of progressive structural damage than synovitis, suggesting that a potential erosive tendency in some patients more strongly influences long-term structural damage outcome than synovial inflammation.

Based on the results of this study, we would recommend routinely performing MRI in ERA patients at baseline to identify at the onset those ERA patients with existing structural damage, indicative of a progressive structural damage pattern. The presence of two distinct structural damage groups suggests a need for two different treatment regimens and treatment targets. It is noteworthy that moderate-severe structural damage was present even at baseline in most patients with a progressive structural damage pattern indicating that these patients are currently being identified too late. The average time interval between symptom onset and recruitment was 10 months, which is similar to other studies [37]. A small number (< 10%) of patients had none-mild structural damage at baseline and year-1, though it progressed to moderate-severe structural damage at year-8.

Our study has some limitations. First, like other MRI-based follow-up ERA studies, only 60% of the initial patient cohort underwent final MRI assessment as patients had left the region, were unwilling to attend for MRI, had developed concurrent illnesses, or were deceased [18, 38–40]. There was no difference in clinical, serological, radiographic, or MRI parameters between patients at baseline who did not complete or who did complete the year-8 follow up (*p* > 0.05 for all). Second, one cannot infer that changes seen in the wrist will necessarily be mirrored in other joints. Third, as biologic DMARDS are not reimbursed by the local health authority, only one-fifth of patients were on biologic DMARDs by year-8, and, as such, the effect of such treatment on long-term structural damage was not evaluated [41, 42].

In conclusion, this eight-year MRI-based study identified two distinct structural damage patterns in ERA patients, namely a “non-progressive structural damage pattern” seen in two-thirds of patients and a “progressive structural damage pattern” seen in one-third of patients. Only baseline MRI, and not clinical, serological, or radiographic assessment, could reliably predict the severity of structural damage at year-8.

## Supplementary information

Supplementary Information

## Abbreviations

AUC: Area under curve
BME: Bone marrow edema
DAS 28: Disease Activity Index 28
DMARDs: Disease-modifying antirheumatic drugs
ERA: Early rheumatoid arthritis
HAQ-DI: Health Assessment Questionnaire
JSN: Joint space narrowing
RAMRIS: Rheumatoid Arthritis Magnetic Resonance Imaging Scoring System
ROC: Receiver operating characteristic
SDAI: Simple Disease Activity Index
SDC: Smallest detectable change
SHS: Sharp-van der Heijde

## References

[1] Perera J, Delrosso CA, Nerviani A, Pitzalis C (2024) Clinical phenotypes, serological biomarkers, and synovial features defining seropositive and seronegative rheumatoid arthritis: a literature review. Cells 13:743 38727279 10.3390/cells13090743 PMC11083059

[2] Buch MH, Eyre S, McGonagle D (2021) Persistent inflammatory and non-inflammatory mechanisms in refractory rheumatoid arthritis. Nat Rev Rheumatol 17:17–33 33293696 10.1038/s41584-020-00541-7

[3] Aletaha D, Smolen JS (2018) Diagnosis and management of rheumatoid arthritis: a review. JAMA 320:1360–1372 30285183 10.1001/jama.2018.13103

[4] Smolen JS, Aletaha D, McInnes IB (2016) Rheumatoid arthritis. (published correction appears in Lancet 22:388 (1984)). Lancet 388:2023–2038 10.1016/S0140-6736(16)30173-8 27156434

[5] Salaffi F, Carotti M, Di Carlo M et al (2024) Magnetic resonance imaging (MRI)-based semi-quantitative methods for rheumatoid arthritis: from scoring to measurement. J Clin Med 13:4137 39064179 10.3390/jcm13144137 PMC11277801

[6] Ørnbjerg LM, Østergaard M (2019) Assessment of structural damage progression in established rheumatoid arthritis by conventional radiography, computed tomography, and magnetic resonance imaging. Best Pract Res Clin Rheumatol 33:101481 32001166 10.1016/j.berh.2019.101481

[7] Lee CH, Srikhum W, Burghardt AJ et al (2015) Correlation of structural abnormalities of the wrist and metacarpophalangeal joints evaluated by high-resolution peripheral quantitative computed tomography, 3 Tesla magnetic resonance imaging and conventional radiographs in rheumatoid arthritis. Int J Rheum Dis 18:628–639 25293500 10.1111/1756-185X.12495

[8] Døhn UM, Conaghan PG, Eshed I et al (2014) The OMERACT-RAMRIS rheumatoid arthritis magnetic resonance imaging joint space narrowing score: intrareader and interreader reliability and agreement with computed tomography and conventional radiography. J Rheumatol 41:392–397 24293568 10.3899/jrheum.131087

[9] Xiao F, Griffith JF, Ko JKL et al (2021) MRI wrist in early rheumatoid arthritis: reduction in inflammation assessed quantitatively during treatment period correlates best with clinical improvement. Skeletal Radiol 50:1337–1345 33244616 10.1007/s00256-020-03669-5

[10] Pialat JB, Burghardt AJ, Sode M, Link TM, Majumdar S (2012) Visual grading of motion induced image degradation in high resolution peripheral computed tomography: impact of image quality on measures of bone density and micro-architecture. Bone 50:111–118 22019605 10.1016/j.bone.2011.10.003

[11] van der Heijde D (2000) How to read radiographs according to the Sharp/van der Heijde method. J Rheumatol 27:261–263 10648051

[12] Østergaard M, Peterfy CG, Bird P et al (2017) The OMERACT Rheumatoid Arthritis Magnetic Resonance Imaging (MRI) Scoring System: Updated Recommendations by the OMERACT MRI in Arthritis Working Group. J Rheumatol 44:1706–1712 28811353 10.3899/jrheum.161433

[13] Yushkevich PA, Piven J, Hazlett HC et al (2006) User-guided 3D active contour segmentation of anatomical structures: significantly improved efficiency and reliability. Neuroimage 31:1116–1128 16545965 10.1016/j.neuroimage.2006.01.015

[14] Hodgson RJ, O’Connor P, Moots R (2008) MRI of rheumatoid arthritis image quantitation for the assessment of disease activity, progression and response to therapy. Rheumatology 47:13–21 18045811 10.1093/rheumatology/kem250

[15] Yiu C, Griffith JF, Xiao F et al (2024) Automated quantification of wrist bone marrow oedema, pre- and post-treatment, in early rheumatoid arthritis. Rheumatol Adv Pract 8:rkae073 38915843 10.1093/rap/rkae073 PMC11194532

[16] Bruynesteyn K, Boers M, Kostense P, van der Linden S, van der Heijde D (2005) Deciding on progression of joint damage in paired films of individual patients: smallest detectable difference or change. Ann Rheum Dis 64:179–182 15286006 10.1136/ard.2003.018457 PMC1755378

[17] McQueen FM, Benton N, Perry D et al (2003) Bone edema scored on magnetic resonance imaging scans of the dominant carpus at presentation predicts radiographic joint damage of the hands and feet six years later in patients with rheumatoid arthritis. Arthritis Rheum 48:1814–1827 12847674 10.1002/art.11162

[18] Glinatsi D, Baker JF, Hetland ML et al (2017) Magnetic resonance imaging assessed inflammation in the wrist is associated with patient-reported physical impairment, global assessment of disease activity and pain in early rheumatoid arthritis: longitudinal results from two randomised controlled trials. Ann Rheum Dis 76:1707–1715 28611080 10.1136/annrheumdis-2017-211315

[19] Kamishima T, Fujieda Y, Atsumi T et al (2010) Contrast-enhanced whole-body joint MRI in patients with unclassified arthritis who develop early rheumatoid arthritis within 2 years: feasibility study and correlation with MRI findings of the hands. AJR Am J Roentgenol 195:W287–W292 20858791 10.2214/AJR.09.4140

[20] Sundin U, Sundlisater NP, Aga AB et al (2021) Value of MRI and ultrasound for prediction of therapeutic response and erosive progression in patients with early rheumatoid arthritis managed by an aggressive treat-to-target strategy. RMD Open 7:e001525 33547228 10.1136/rmdopen-2020-001525 PMC7871342

[21] Baker JF, Conaghan PG, Emery P, Baker DG, Ostergaard M (2017) Relationship of patient-reported outcomes with MRI measures in rheumatoid arthritis. Ann Rheum Dis 76:486–490 27432355 10.1136/annrheumdis-2016-209463

[22] Glinatsi D, Brahe CH, Hetland ML et al (2020) Association between MRI findings and patient-reported outcomes in patients with rheumatoid arthritis in clinical remission and at relapse. Int J Rheum Dis 23:488–498 31994328 10.1111/1756-185X.13790

[23] Eberhard A, Rydell E, Forslind K et al (2023) Radiographic damage in early rheumatoid arthritis is associated with increased disability but not with pain-a 5-year follow-up study. Arthritis Res Ther 25:29 36849881 10.1186/s13075-023-03015-9 PMC9969673

[24] Zhang H, Xu H, Chen S, Mao X (2018) The application value of MRI in the diagnosis of subclinical inflammation in patients with rheumatoid arthritis in remission. J Orthop Surg Res 13:164 29970124 10.1186/s13018-018-0866-2 PMC6029344

[25] Rydell E, Forslind K, Nilsson JÅ, Jacobsson LTH, Turesson C (2018) Smoking, body mass index, disease activity, and the risk of rapid radiographic progression in patients with early rheumatoid arthritis. Arthritis Res Ther 20:82 29720260 10.1186/s13075-018-1575-2 PMC5932864

[26] Legrand J, Kirchgesner T, Sokolova T, Vande Berg B, Durez P (2019) Early clinical response and long-term radiographic progression in recent-onset rheumatoid arthritis: Clinical remission within six months remains the treatment target. Joint Bone Spine 86:594–599 30928534 10.1016/j.jbspin.2019.03.008

[27] Svensson B, Andersson MLE, Gjertsson I, Hafström I, Ajeganova S, Forslind K (2022) Erosion-free rheumatoid arthritis: clinical and conceptional implications-a BARFOT study. BMC Rheumatol 6:88 36581910 10.1186/s41927-022-00317-4 PMC9801569

[28] Tamai M, Arima K, Nakashima Y et al (2017) Baseline MRI bone erosion predicts the subsequent radiographic progression in early rheumatoid arthritis patients who achieved sustained good clinical response. Mod Rheumatol 27:961–966 28269999 10.1080/14397595.2017.1294280

[29] Fukae J, Tanimura K, Isobe M et al (2018) Active synovitis in the presence of osteitis predicts residual synovitis in patients with rheumatoid arthritis with a clinical response to treatment. Int J Rheum Dis 21:1809–1814 28160411 10.1111/1756-185X.13030

[30] Møller-Bisgaard S, Hørslev-Petersen K, Ørnbjerg LM et al (2024) Long-term efficacy of a 2-year MRI treat-to-target strategy on disease activity and radiographic progression in patients with rheumatoid arthritis in clinical remission: 5-year follow-up of the IMAGINE-RA randomised trial. RMD Open 10:e003945 38490697 10.1136/rmdopen-2023-003945 PMC10946351

[31] Rivellese F, Humby F, Bugatti S et al (2020) B Cell Synovitis and Clinical Phenotypes in Rheumatoid Arthritis: Relationship to Disease Stages and Drug Exposure. Arthritis Rheumatol 72:714–725 31785084 10.1002/art.41184 PMC7217046

[32] Humby F, Durez P, Buch MH et al (2021) Rituximab versus tocilizumab in anti-TNF inadequate responder patients with rheumatoid arthritis (R4RA): 16-week outcomes of a stratified, biopsy-driven, multicentre, open-label, phase 4 randomised controlled trial. Lancet 397:305–317 33485455 10.1016/S0140-6736(20)32341-2 PMC7829614

[33] Conaghan PG, Østergaard M, Troum O et al (2019) Very early MRI responses to therapy as a predictor of later radiographic progression in early rheumatoid arthritis. Arthritis Res Ther 21:214 31639034 10.1186/s13075-019-2000-1 PMC6805378

[34] Ramírez J, Cuervo A, Celis R et al (2021) Biomarkers for treatment change and radiographic progression in patients with rheumatoid arthritis in remission: a 5-year follow-up study. Rheumatology (Oxford) 60:667–674 32653929 10.1093/rheumatology/keaa258

[35] van Nies JA, van Steenbergen HW, Krabben A et al (2015) Evaluating processes underlying the predictive value of baseline erosions for future radiological damage in early rheumatoid arthritis. Ann Rheum Dis 74:883–889 24431393 10.1136/annrheumdis-2013-204659

[36] Suter LG, Fraenkel L, Braithwaite RS (2011) Role of magnetic resonance imaging in the diagnosis and prognosis of rheumatoid arthritis. Arthritis Care Res (Hoboken) 63:675–688 21557523 10.1002/acr.20409 PMC3135707

[37] Barhamain AS, Magliah RF, Shaheen MH et al (2017) The journey of rheumatoid arthritis patients: a review of reported lag times from the onset of symptoms. Open Access Rheumatol 9:139–150 28814904 10.2147/OARRR.S138830 PMC5546831

[38] Ahmad HA, Baker JF, Østergaard M, Ye J, Emery P, Conaghan PG (2019) Determining MRI Inflammation Targets When Considering a Rheumatoid Arthritis Treat-to-Target Strategy: Results of a Randomized, Placebo-Controlled Trial. Adv Ther 36:2384–2393 31278695 10.1007/s12325-019-01020-6 PMC6822846

[39] Boer AC, Wouters F, Dakkak YJ, Niemantsverdriet E, van der Helm-van Mil AHM (2020) Improving the feasibility of MRI in clinically suspect arthralgia for prediction of rheumatoid arthritis by omitting scanning of the feet. Rheumatology 59:1247–1252 31566238 10.1093/rheumatology/kez436 PMC7244779

[40] Hetland ML, Østergaard M, Stengaard-Pedersen K et al (2019) Anti-cyclic citrullinated peptide antibodies, 28-joint Disease Activity Score, and magnetic resonance imaging bone oedema at baseline predict 11 years’ functional and radiographic outcome in early rheumatoid arthritis. Scand J Rheumatol 48:1–8 30101636 10.1080/03009742.2018.1466362

[41] Lin YJ, Anzaghe M, Schülke S (2020) Update on the Pathomechanism, Diagnosis, and Treatment Options for Rheumatoid Arthritis. Cells 9:880 32260219 10.3390/cells9040880 PMC7226834

[42] Ishiguro N, Tanaka Y, Yamanaka H et al (2019) Efficacy of denosumab with regard to bone destruction in prognostic subgroups of Japanese rheumatoid arthritis patients from the phase II DRIVE study. Rheumatology (Oxford) 58:997–1005 30602032 10.1093/rheumatology/key416 PMC6532444

