# Supplementary material for: Early rheumatoid arthritis, two distinctive structural damage patterns revealed by MRI: an 8-year longitudinal study
Source: Eur Radiol. 2025 Mar 18;35(9):5403–17. doi: 10.1007/s00330-025-11493-5 (PMC12350573; doi:10.1007/s00330-025-11493-5)
Supplement: Supplementary file 1 — Supplementary Information [file 330_2025_11493_MOESM1_ESM.pdf]

Supplemental material 1:

MRI scanning protocol:

Wrists were scanned in a Superman position on a 3.0-T system (Phillips Integra, Best, Netherlands) using a dedicated wrist coil and a scan plane extending from the distal radio-ulnar joints to the metacarpal bases. The following sequences were obtained: fat-saturated T1-weighted axial (TR 692ms; TE 12ms; echo spacing 12ms; flip angle 90°; FOV 80×80mm; slice thickness 3mm; NEX 2); fat-saturated T2-weighted coronal (TR 3121ms; TE 70ms; echo spacing 10ms; flip angle 90°; FOV 80×80mm; slice thickness 1.5mm; NEX 1); T1-weighted coronal (TR 547ms; TE 12ms; echo spacing 12ms; flip angle 90°; FOV 80×80mm; slice thickness 1.5mm; NEX 1); fat-saturated post-contrast T1-weighted axial (TR 692ms; TE 12ms; echo spacing 12ms; flip angle 90°; FOV 80×80mm; slice thickness 3mm; NEX 2). Dynamic contrast enhanced MRI (DCE MRI) was also performed (TR 3.8ms; TE 2.3ms; echo spacing 59.7ms; flip angle 12°; FOV 100 × 82mm; slice thickness 5mm; NEX 2) utilizing a pump injector and intravenous gadolinium contrast dose of 0.1mmol/kg.

Supplemental figure 1:

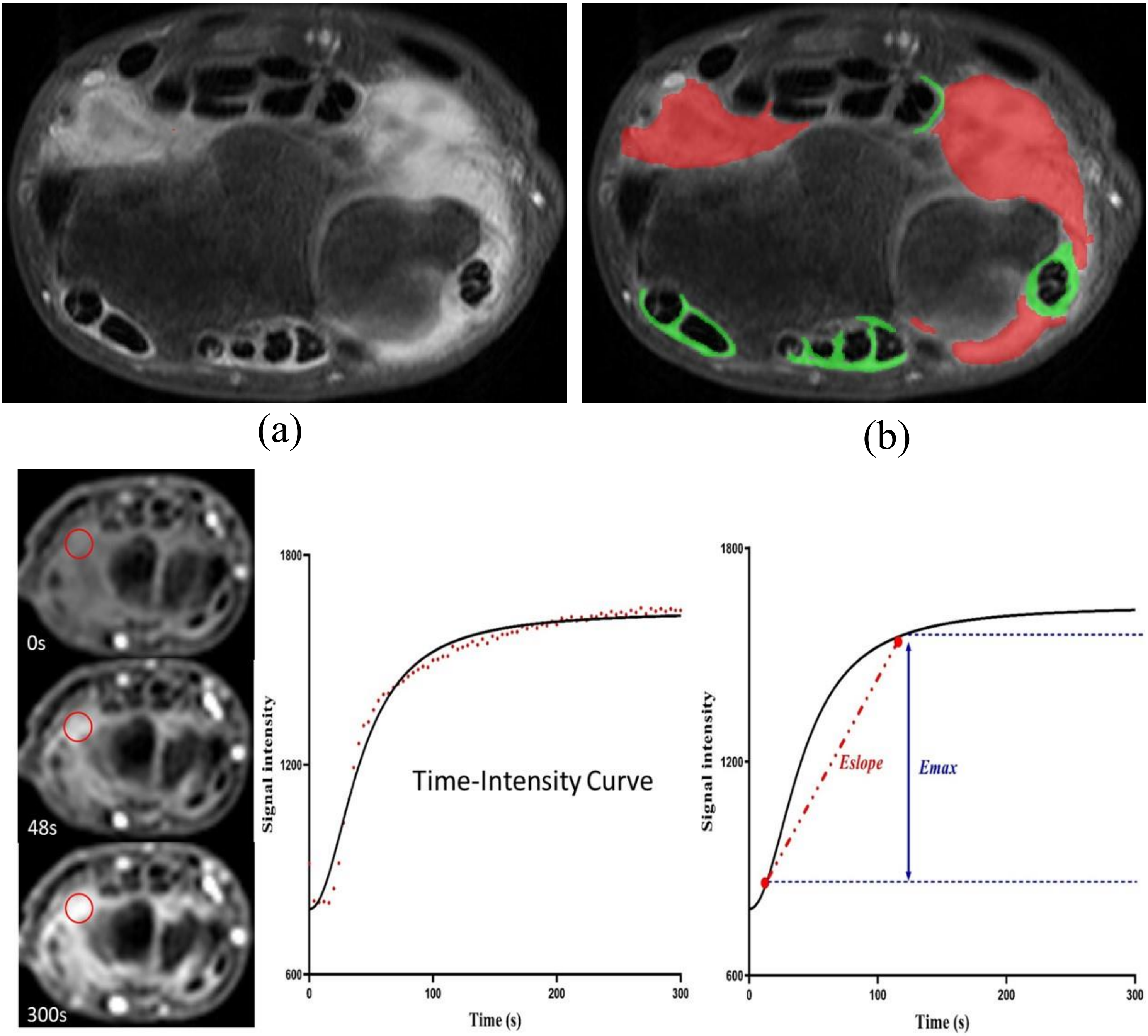

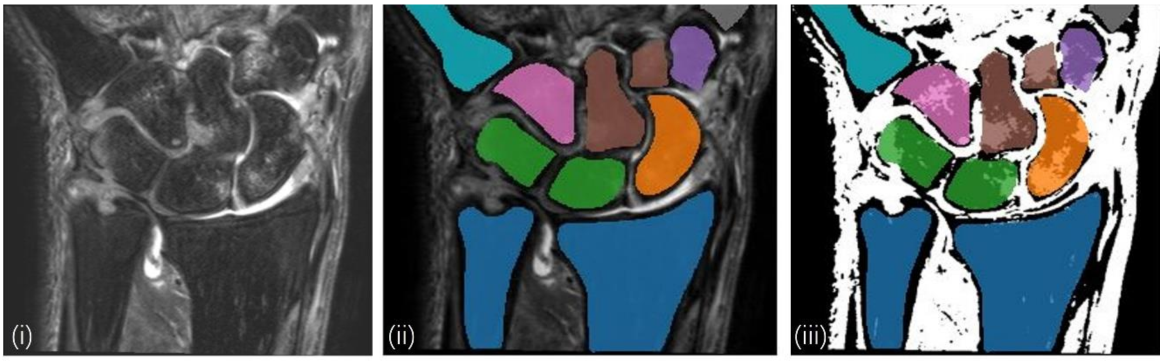

	BME proportion	Intensity (mean)	Intensity (sd)
Overall	22.1	4.6	2.6
Carpal	33.4	4.5	2.4
Radius	6.2	2.9	0.9
Ulna	1.9	3.4	1.5
Scaphoid	28.7	4.2	2.3
Lunate	27.1	4.3	2.3

(c)

Supplemental figure 1: (a) 61-year-old female with severe wrist synovitis and mild tenosynovitis on post-contrast axial T1-weighted MR images with fat-suppression. (b) Regions of synovitis (red) and tenosynovitis (green) were manually segmented on serial images and summated. Synovitis volume: 14.6 cm^3; tenosynovitis volume: 2.7 cm^3 (c): 75-year-old female with moderate synovitis. Red dots represent the mean signal intensity of the red circled region of interest at different timepoints. The black curve is the fitted time-intensity curve (TIC). Emax and Eslope were calculated from the TIC as shown. (d): 67-year-old male with moderate-severity bone marrow edema. (i) After the T2FS coronal MR images were uploaded to the program, (ii) wrist bones are automatically segmented and (iii) the proportion (%) and relative intensity of BME automatically calculated. Overall BME proportion for this patient was 22.1% and BME relative intensity was 4.7± 2.6. BME: bone marrow edema. sd: standard deviation.

Supplemental figure 2:

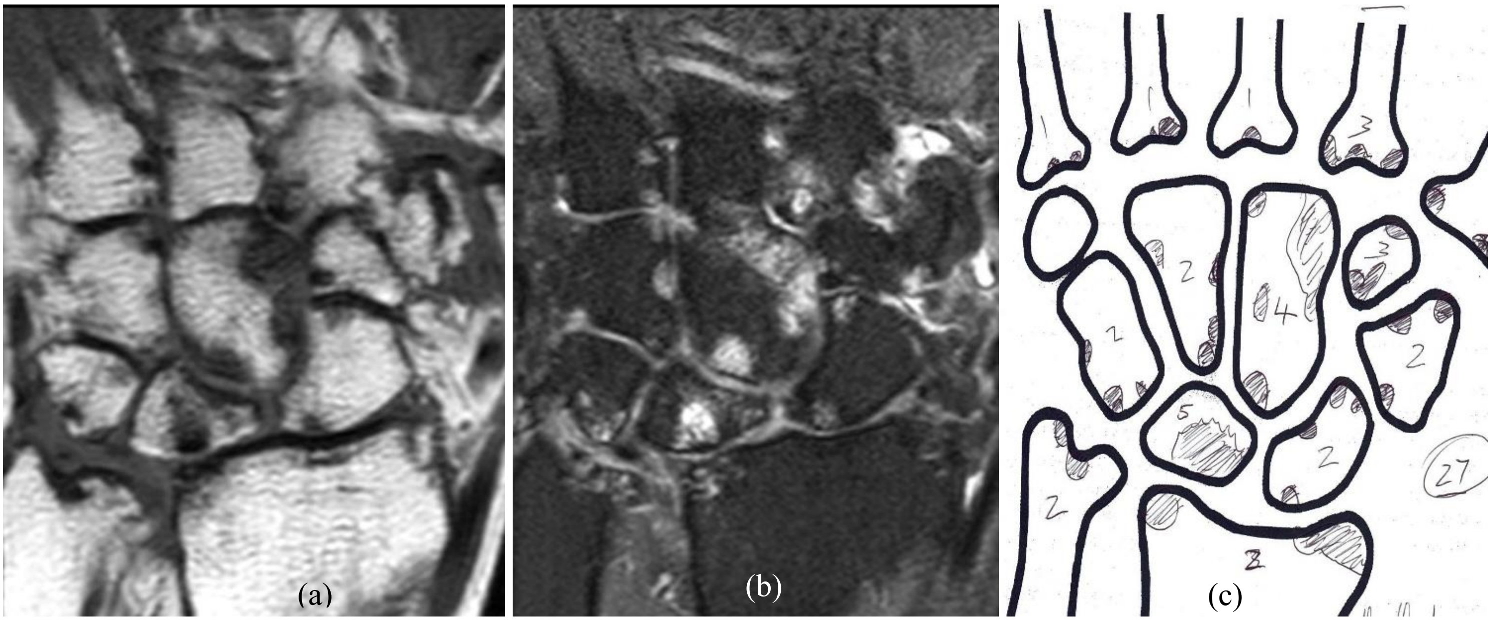

Supplemental figure 2: Adult patient at year-8 follow-up. (a) T1WI coronal image, (b) T2W fat suppressed coronal image. (c) Schematic template with location and size of bone erosions labelled. Erosions were marked based on assessment of all images rather than a single image. Total erosion score for this patient was 27.

Supplemental Table 1:

Supplementary table 1: Inter- and intra- class correlation coefficient results of synovitis, bone marrow edema, JSN, bone erosion and total score (n=145)

	Synovitis	Teno-synovitis	BME	Bone erosion	JSN	Total score
Inter-class correlation	0.833	0.910	0.900	0.921	0.938	0.926
Intra-class correlation						
Reader 1	0.894	0.908	0.854	0.864	0.880	0.945
Reader 2	0.909	0.941	0.900	0.921	0.895	0.961
